# The effect of IV fluid supplementation and feeding method on bilirubin elimination in term neonates with severe indirect hyperbilirubinemia: a retrospective cohort study

**DOI:** 10.3389/fped.2026.1868732

**Published:** 2026-06-03

**Authors:** Zeynep Tobcu, Begum Baris Cetinkaya, Yasin Selcuk Yardibi, Sevim Orum, Dondu Ulker Ustebay, Rulin Deniz, Yakup Baykus, Alihan Tigli, Guzide Ece Akinci, Muhammet Bora Uzuner, Sefer Ustebay

**Affiliations:** 1 Department of Pediatrics, Faculty of Medicine, Bandırma Onyedi Eylül University, Bandirma, Türkiye; 2 Department of Pediatric Neurology, Faculty of Medicine, Bandırma Onyedi Eylül University, Bandirma, Türkiye; 3 Department of Obstetrics and Gynecology, Faculty of Medicine, Bandırma Onyedi Eylül University, Bandirma, Türkiye; 4 Gynecology and Obstetrics Clinic, Şehit Prof. Dr. İlhan Varank Sancaktepe Training and Research Hospital, Istanbul, Türkiye; 5 Department of Anatomy, Faculty of Medicine, Bandırma Onyedi Eylül University, Bandirma, Türkiye

**Keywords:** bilirubin elimination, breast milk, enteral feeding, indirect hyperbilirubinemia, intensive phototherapy, intravenous fluids, neonatal jaundice

## Abstract

**Background:**

This study aimed to assess the independent effects of intravenous (IV) fluid supplementation and different enteral feeding patterns on bilirubin elimination in healthy term neonates treated with intensive phototherapy for severe indirect hyperbilirubinemia.

**Materials and methods:**

This single-center, retrospective cohort study included 271 healthy term newborns who received phototherapy for indirect hyperbilirubinemia between 2023 and 2025. The neonates were divided into two groups: those receiving enteral feeding alone (*n* = 160) and those receiving IV fluid supplementation in addition to enteral feeding (*n* = 111). Data were analyzed using multivariate linear regression supported by sensitivity analyses adjusting for additional maternal and neonatal confounders, to account for baseline clinical differences and potential indication bias.

**Results:**

The IV fluid supplementation group had significantly higher baseline total serum bilirubin (TSB) levels and postnatal age than the enteral feeding-only group (*p* < 0.001). After adjustment for baseline clinical differences, IV fluid supplementation was not independently associated with the rate or the percentage of bilirubin decline (*p* > 0.05). In contrast, mixed feeding with formula supplementation in addition to breastfeeding and a high baseline TSB level were independently associated with faster bilirubin clearance (*p* < 0.001). Higher maternal age was independently associated with a greater percentage reduction in bilirubin levels (*p* = 0.018).

**Conclusion:**

In term neonates with severe indirect hyperbilirubinemia with no clinical evidence of dehydration, IV fluid supplementation appeared to provided no additional benefit in reducing bilirubin levels. Our findings suggest that enteral feeding patterns may have a greater influence on the response to phototherapy. Therefore, our findings suggest that routine IV fluid administration may not be necessary in the management of hyperbilirubinemia, and priority should be given to effective lactation support and enteral feeding. When breast milk intake is insufficient, formula supplementation should be considered as a temporary and supportive option to accelerate bilirubin elimination, however, due to the heterogeneity in feeding volumes, this effect should be interpreted cautiously.

## 1 Introduction

Neonatal jaundice is one of the most frequently encountered clinical conditions in the neonatal period and, is usually physiological, transient and associated with a favorable prognosis (1). Nevertheless, severe indirect hyperbilirubinemia may develop in some neonates and can result in serious complications, including acute bilirubin encephalopathy and kernicterus, due to the neurotoxic effects of bilirubin (2). For this reason, timely and effective management is crucial, particularly in cases where total serum bilirubin (TSB) levels reach treatment thresholds (3).

Phototherapy remains the cornerstone of treatment for severe indirect hyperbilirubinemia. It converts bilirubin into more water-soluble photoisomers that can be eliminated via urine and stool. However, phototherapy may be associated with several adverse effects, such as increased transdermal fluid loss, hyperthermia, diarrhea, skin rash, reduced mother-infant contact, and disruption of feeding patterns (4). Accordingly, ensuring adequate hydration in neonates receiving phototherapy is regarded as an essential component of clinical management.

Current guidelines from the American Academy of Pediatrics (AAP) do not recommend routine intravenous (IV) fluid supplementation for neonates receiving phototherapy for hyperbilirubinemia. Instead, they emphasize maintaining adequate enteral feeding and appropriate hydration. IV fluid administration is recommended only in selected cases, such as clinical dehydration, inadequate oral intake, or borderline/severe hyperbilirubinemia (5, 6). Consistent with this, several studies report that routine IV fluid supplementation in healthy term neonates does not significantly accelerate bilirubin decline or provide a clinically significant reduction in phototherapy duration (7–13). Nevertheless, IV fluid supplementation remains widely used in clinical practice, primarily with the aim of promoting a more rapid bilirubin decrease and mitigating concerns regarding inadequate oral intake.

Beyond the total volume of fluid intake, the mode of enteral feeding may also influence bilirubin metabolism in neonates. Frequent and effective enteral feeding promotes intestinal motility, reduces the enterohepatic circulation of bilirubin, and facilitates bilirubin elimination (14). Mixed feeding, particularly when formula is used to supplement breast milk, has been reported to reduce the enterohepatic circulation more effectively than exclusive breastfeeding and may thereby accelerate the decline in bilirubin levels (15, 16). However, this approach requires careful consideration of the clinical balance between short-term bilirubin control and the long-term continuation of breastfeeding (17).

Exchange transfusion is reserved for cases in which indirect hyperbilirubinemia cannot be adequately controlled and represents an invasive procedure associated with serious complications including infection, apnea, anemia, electrolyte imbalances, and embolism. Therefore, identifying supportive approaches that are easy to implement, less invasive, and capable of enhancing the efficacy of phototherapy is clinically critical.

Although current evidence suggests limited benefit from routine IV fluid supplementation, prior observational studies have frequently been limited by residual confounding due to indication bias and the failure to simultaneously evaluate the impact of different enteral feeding modalities. To address this specific methodological gap, few studies have examined the independent effects of IV fluid supplementation and enteral feeding patterns on bilirubin decline within the same cohort of healthy term neonates undergoing intensive phototherapy in the absence of clinical dehydration. Therefore, the primary aim of this study is to compare the rates and percentage of serum bilirubin decline between healthy term neonates with severe indirect hyperbilirubinemia receiving enteral feeding alone and those receiving IV fluid supplementation in addition to enteral feeding. The secondary aim is to evaluate the independent effects of different enteral feeding patterns, such as exclusive breastfeeding and mixed feeding, on bilirubin clearance.

## 2 Materials and methods

### 2.1 Study design and patient selection

This study was designed as a single-center retrospective cohort study and has been reported in accordance with the Strengthening the Reporting of Observational Studies in Epidemiology (STROBE) guidelines. As part of the study, the medical records of a total of 400 neonates who received phototherapy for indirect hyperbilirubinemia in the Level II neonatal intensive care unit of a tertiary university hospital between 2023 and 2025 were retrospectively reviewed. In our unit, patient flow and clinical management are standardized, with all neonates managed under a uniform institutional protocol based on the AAP guidelines. This standardization ensured that phototherapy indications, targeted enteral feeding frequencies, fluid administration calculations, and monitoring of TSB and hydration status were consistently applied to all patients, thereby minimizing variations in clinical practice.

**Figure 1:**
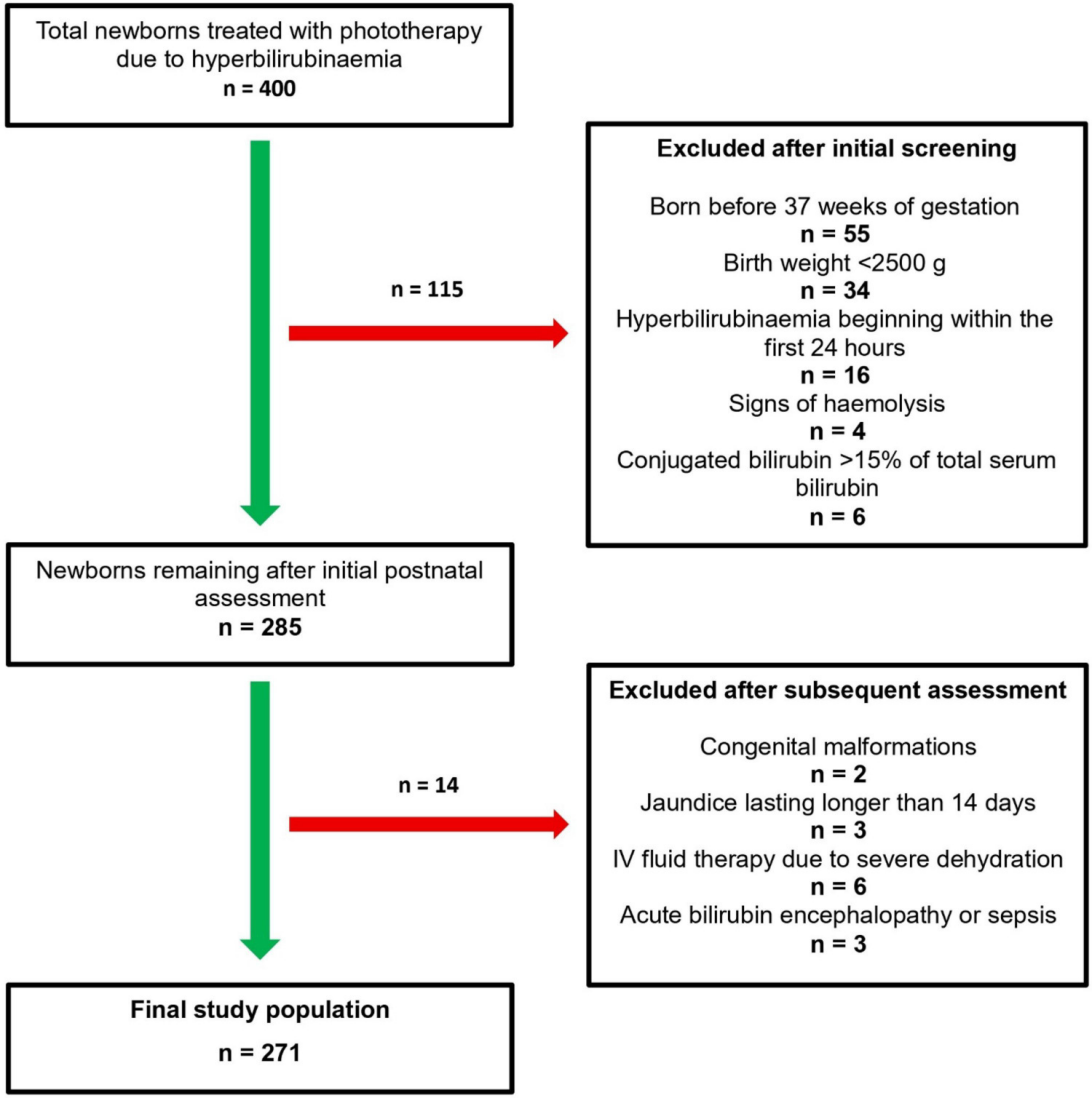
Flowchart of the study.

The inclusion criteria for the study were: gestational age of at least 37 weeks, birth weight of at least 2,500 g, jaundice commencing after the first 24 h of life, absence of hemolysis, and a conjugated bilirubin level below 15% of the total serum bilirubin (TSB) level. The absence of hemolysis was defined by a negative direct Coombs test and a reticulocyte count below 6%. Neonates with a birth weight below 2,500 g, those with acute bilirubin encephalopathy or signs of significant dehydration, those with major congenital malformations, those with jaundice lasting longer than 14 days, those who had received intravenous fluid supplementation prior to admission to the clinic, or those diagnosed with sepsis were excluded from the study. Due to the retrospective nature of the study, an *a priori* sample size calculation was not performed; instead, all eligible consecutive neonates treated during the study period were included. As a result of these criteria, a total of 271 neonates were included in the study. Cases were divided into two main groups according to the treatment approach: those receiving enteral feeding alone (*n* = 160) and those receiving IV fluid supplementation in addition to enteral feeding (*n* = 111) (Figure 1).

### 2.2 Phototherapy treatment and clinical follow-up

Intensive phototherapy was administered bilaterally using a narrow-spectrum blue LED light source with a wavelength of 460–490 nm, to provide a light intensity of at least 30 µW/cm^2^/nm on the skin surface. The indication for phototherapy was determined in accordance with AAP recommendations, considering postnatal age, gestational age and TSB level. Short interruptions were allowed during phototherapy to support breastfeeding and maintain mother-infant interaction, with each interruption not exceeding 30 min. All neonates were targeted to receive enteral feeding at least 8–12 times per day and received enteral feeding before phototherapy was initiated. To assess the early response to phototherapy, a control TSB level was measured at the 6th hour of treatment (5, 6, 18, 19).

### 2.3 Fluid supplementation and nutritional management

The decision to initiate IV fluid supplementation was made by the attending pediatricians in our unit, based on clinical criteria such as the infant's clinical hydration status, a history of inadequate oral intake, or the presence of borderline/severe hyperbilirubinemia. Randomization was not performed, and this treatment decision was based entirely on individualized clinical assessments. In neonates receiving intravenous fluid supplementation, 25% of the calculated total daily fluid requirement was administered intravenously. The total daily fluid requirement was calculated in accordance with approaches recommended as 80 mL/kg/day for day 2, 100 mL/kg/day for day 3, and 150 mL/kg/day from day 4 onwards. Additional IV fluids were administered as a continuous infusion of a 1:5 solution of normal saline and 5% dextrose which was initiated concurrently with the commencement of phototherapy (16, 20). To prevent excessive hydration during treatment, the patients' hydration status, fluid balance, and TSB levels, serum sodium, urea and creatinine were closely monitored. All cases were analyzed in four subgroups based on both IV fluid status and feeding type to assess the combined effect of the treatment approach on clinical outcomes: exclusive breastfeeding, mixed feeding, IV fluids + breastfeeding, and IV fluids + mixed feeding. Feeding type was categorized as exclusive breastfeeding and mixed feeding. As net enteral volume data were unavailable, mixed feeding was defined as cases receiving formula milk supplementation at any frequency in addition to breast milk, regardless of the amount. Enteral feeding was provided to all neonates prior to phototherapy.

### 2.4 Data collection, outcomes and statistical analysis

Demographic and clinical data were obtained retrospectively from medical records. The variables collected included gestational age, birth weight, sex, mode of delivery, postnatal age, maternal age, hemoglobin level, length of hospital stay, baseline TSB level, and TSB level at the 6th hour of phototherapy.

The primary endpoints of this study were the rate and the percentage of bilirubin decline. The rate of decline was calculated as (baseline TSB−6-h TSB) divided by the elapsed time, whereas the percentage decline was calculated as [(baseline TSB−6-h TSB)/baseline TSB] × 100. The 6-h TSB level was evaluated as a secondary endpoint.

Statistical analyses were performed using IBM SPSS Statistics for Windows, version 23.0. Categorical variables were presented as counts and percentages, whilst continuous variables were presented as mean ± standard deviation or median [interquartile range (IQR)] according to their distribution characteristics. The chi-square test or Fisher's exact test was used to compare categorical data. After assessing the assumption of normal distribution for continuous variables, the independent samples t-test or Mann–Whitney *U*-test was applied for comparisons between two groups; for comparisons involving three or more groups, one-way analysis of variance (ANOVA) or the Kruskal–Wallis test was applied. Relationships between continuous variables were examined using Pearson or Spearman correlation analysis, depending on the distribution characteristics.

Extended multivariate linear regression models were constructed to control for potential indication bias arising from differences in baseline TSB levels and postnatal age observed between groups. In these models, the dependent variables were defined as the rate of bilirubin decline, the percentage of bilirubin decline, and the TSB level at 6 h. The main independent variables were identified as the presence of IV fluid supplementation and the type of enteral feeding. In addition to the baseline model, potential confounding variables such as hemoglobin level, maternal age, gestational age, birth weight, and mode of delivery were included in the model as part of sensitivity analyses. These variables were selected to isolate confounding effects that may be associated with bilirubin kinetics in the literature and may arise from baseline differences between groups. Furthermore, an interaction term was added to the regression models to assess whether there was a synergistic interaction between IV fluid supplementation and the type of enteral nutrition. In all analyses, a two-sided *p*-value below 0.05 was considered statistically significant.

Prior to interpretation of the multivariable linear regression models, model assumptions were evaluated. The normality of standardized residuals was assessed by visual inspection of histograms and normal probability plots. Homoscedasticity was evaluated using scatterplots of standardized residuals against standardized predicted values. Multicollinearity was assessed using tolerance and variance inflation factor values, with tolerance values >0.20 and variance inflation factor values <5 considered acceptable. No problematic multicollinearity was detected in the final models. There were no missing data for the variables included in the final regression models; therefore, no imputation procedure was required. Covariates were selected *a priori* based on clinical relevance, biological plausibility, previous literature, and observed baseline differences between treatment groups.

## 3 Results

### 3.1 Demographic and clinical characteristics

A total of 271 term neonates who met the inclusion criteria were included in the study. The cases were divided into two main groups based on the treatment approach: those receiving enteral feeding alone (*n* = 160, 59%) and those receiving IV fluid supplementation in addition to enteral feeding (*n* = 111, 41%).

No statistically significant differences were observed between the two groups in terms of gender distribution, mode of delivery, birth weight, gestational age, maternal age, parity, and baseline hemoglobin level (*p* > 0.05).

In contrast, the groups differed significantly in several baseline clinical characteristics. The IV fluid supplementation group had higher baseline TSB levels than the enteral feeding-alone group (median, 18.10 mg/dL vs. 15.95 mg/dL; *p* < 0.001). Postnatal age was also higher in this group (median, 4 days vs. 3 days; *p* = 0.001).

When assessed in terms of feeding type, the rate of mixed feeding was higher among neonates receiving IV fluid supplementation. In this group, 64% of cases (*n* = 71) received formula milk in addition to breast milk, whereas this rate was only 30% (*n* = 48) in the group receiving enteral feeding alone (*p* < 0.001). Detailed demographic and clinical characteristics of the patients are presented in Table 1.

**Table 1:** Demographic and clinical characteristics of neonates according to IV fluid supplementation Status.

Variable	IV Fluid Therapy (‒)	IV Fluid Therapy (+)	Test statistic	*p* *
	(*n* = 160)	(*n* = 111)		
	*n* (%)	*n* (%)		
Sex			*χ*^2^ = 0.026	0.871
Male	82 (51.2)	58 (52.3)		
Female	78 (48.8)	53 (47.7)		
Delivery mode			χ^2^ = 0.239	0.625
NSD	74 (46.2)	48 (43.2)		
C/S	86 (53.8)	63 (56.8)		
Oral feeding type			χ^2^ = 30.694	<0.001
Breast milk only	112 (70.0)	40 (36.0)		
Breast milk only and Formula	48 (30.0)	71 (64.0)		
	mean ± SD	mean ± SD		
Hemoglobin	17.30 ± 2.28	17.21 ± 2.33	*t* = 0.324	0.746
	median (IQR)	median (IQR)		
Maternal age (years)	27 (6)	28 (8)	Z = −1.852	0.064
Gravidity	1 (2)	1 (1)	Z = −0.788	0.431
Initial bilirubin, mg/dL	15.95 (5.40)	18.10 (3.68)	Z = −5.081	<0.001
Postnatal age (days)	3 (3)	4 (5)	Z = −3.353	0.001
Birth weight, (g)	3,195 (536)	3,150 (590)	Z = −0.086	0.932
Gestational age (weeks)	38 (1)	38 (1)	Z = −1.006	0.314

SD, standard deviation; IQR, interquartile range; NSD, normal spontaneous delivery; C/S, cesarean section; IV, intravenous; χ^2^, chi-square statistic; t, Student's *t*-test statistic; Z, standardized test statistic for the Mann–Whitney *U*-test.

* Data are presented as mean ± standard deviation, median (interquartile range) or *n* (%) where appropriate. Continuous variables were compared using the independent samples t-test for data showing a normal distribution and the Mann–Whitney *U*-test for data not showing a normal distribution. Categorical variables were compared using the Pearson chi-square test. *p*-value < 0.05 was considered statistically significant.

### 3.2 Bilirubin response according to treatment and dietary group

In the unadjusted comparison, the TSB level measured at the 6th hour of phototherapy was higher in the IV fluid supplementation group than in the enteral feeding-alone group (median, 12.60 mg/dL vs. 10.40 mg/dL; *p* < 0.001).

However, when clinical outcomes that more directly reflecting treatment efficacy were evaluated, no significant differences were observed between the two main groups. The IV fluid supplementation and enteral feeding-alone groups were found to be similar in terms of the hourly rate of bilirubin decline (*p* = 0.250) and the percentage decrease in bilirubin levels (*p* = 0.051). No statistically significant difference was observed between the groups in length of hospital stay (*p* = 0.267). Raw clinical outcome comparisons based on IV fluid supplementation are shown in Table 2.

**Table 2:** Comparison of patient outcomes based on intravenous fluid therapy.

Variable	IV Fluid Therapy (‒)	IV Fluid Therapy (+)	Test statistic	*p* *
	(*n* = 160)	(*n* = 111)		
	median (IQR)	median (IQR)		
6-h bilirubin, mg/dL	10.40 (4.25)	12.60 (3.40)	Z = −4.263	<0.001
Bilirubin decline rate	0.84167 (0.392)	0.88333 (0.383)	Z = −1.151	0.250
Bilirubin decline percentage, %	33.68 (16.23)	30.10 (11.51)	Z = −1.952	0.051
Total hospitalization time (hours)	56.5 (10)	55.0 (13)	Z = −1.109	0.267

IQR, interquartile range; IV, intravenous; Z, standardized test statistic for the Mann–Whitney *U*-test.

* Values are expressed as median (IQR). Group comparisons were performed using the Mann–Whitney *U*-test. A *p* value < 0.05 was considered statistically significant.

To assess the clinical response in greater detail, the cases were analyzed in four subgroups based on their intravenous fluid supplementation status and feeding type: exclusive breastfeeding, mixed feeding, IV fluid supplementation and breastfeeding, and IV fluid supplementation and mixed feeding. Unadjusted analyses across the four groups showed significant differences in both the rate of bilirubin decline (*p* < 0.001) and the percentage of bilirubin decline (*p* = 0.029). These findings, based on feeding type and IV fluid combinations, are presented in Table 3.

**Table 3:** A comparison of the rate of bilirubin decline and the percentage reduction in bilirubin levels according to feeding type and supplementary IV fluid supplementation.

Variable	Group	*N*	Mean rank	χ^2^	*p* *
Bilirubin decline rate	BM	112	111.77	32.406	<0.001
	MF	48	177.33		
	IV + BM	40	115.34		
	IV + MF	71	157.92		
Bilirubin decline percentage	BM	112	135.73	9.000	0.029
	MF	48	162.44		
	IV + BM	40	113.60		
	IV + MF	71	131.18		

BM, breast milk only; MF, mixed/formula-containing feeding; IV, intravenous; χ^2^, Kruskal–Wallis test statistic; p*, probability value.

* Overall group comparisons were performed using the Kruskal–Wallis test. Higher mean ranks indicate relatively higher values of the corresponding outcome.

### 3.3 Factors independently associated with bilirubin elimination

Multivariate linear regression models were constructed to mitigate the effect of clinical differences observed between groups at baseline. In the model established for the 6-h TSB level, the baseline TSB level and the type of enteral nutrition were found to be independently associated variables; however, no statistically significant independent association was detected between IV fluid supplementation and the 6-h TSB level (*p* = 0.269) (Table 4).

**Table 4:** Multivariable linear regression analysis for 6-h bilirubin level.

Variable	B	95% CI	*p*
IV fluid therapy	0.227	−0.176 to 0.629	0.269
Initial bilirubin level	0.841	0.734 to 0.949	<0.001
Oral feeding type	−0.883	−1.307 to −0.458	<0.001

B, unstandardized regression coefficient; CI, confidence interval; IV, intravenous; p, statistical significance level.

Similarly, in the extended regression models established for the hourly bilirubin decline rate and the percentage of bilirubin decline, IV fluid supplementation was not found to be independently significant. In contrast, mixed feeding—combining breast milk with formula—and a higher baseline TSB level were found to be independently associated with both a faster bilirubin decline and a higher percentage decline. IV fluid supplementation, however, showed no independent association with the rate of bilirubin decline (*p* = 0.819) or the percentage of bilirubin decline (*p* = 0.841).

An interaction term was added to the model to assess whether there was a potential interaction between IV fluid supplementation and the type of enteral nutrition; however, no statistically significant interaction was detected. This finding suggests that the relationship between the type of enteral nutrition and the bilirubin response did not change significantly depending on the presence of IV fluid supplementation. Among the other variables included in the model, birth weight and postnatal age were not found to be significant, whereas maternal age showed a positive independent association in both models (*p* = 0.014) (Table 5).

**Table 5:** Extended multivariable linear regression analyses of factors associated with bilirubin decline rate and bilirubin decline percentage.

Variable	Bilirubin Decline Rate				Bilirubin Decline Percentage			
	B (SE)	*β*	95% CI	*p* *	B (SE)	*β*	95% CI	*p* ^†^
Oral feeding type	0.177 (0.046)	0.316	0.085 to 0.268	<0.001	5.915 (1.732)	0.293	2.505 to 9.325	0.001
Initial bilirubin level	0.024 (0.009)	0.250	0.006 to 0.042	0.010	−1.310 (0.341)	−0.382	−1.982 to −0.639	<0.001
Maternal age	0.007 (0.003)	0.133	0.001 to 0.012	0.020	0.262 (0.106)	0.145	0.053 to 0.472	0.014
IV fluid therapy	−0.011 (0.048)	−0.019	−0.105 to 0.083	0.819	−0.358 (1.780)	−0.018	−3.863 to 3.147	0.841
Birth weight	0.000007 (0.000)	0.011	0.000 to 0.000	0.844	0.000 (0.001)	0.010	−0.002 to 0.003	0.858
IV × OFT	−0.066 (0.067)	−0.104	−0.197 to 0.066	0.325	−2.237 (2.495)	−0.098	−7.149 to 2.675	0.371
Postnatal age	−0.004 (0.012)	−0.034	−0.028 to 0.019	0.714	0.048 (0.447)	0.010	−0.832 to 0.929	0.914
**Model statistics**	R = 0.434, R^2^ = 0.189, adjusted R^2^ = 0.167				R = 0.368, R^2^ = 0.135, adjusted R^2^ = 0.112,			
	SEE = 0.253, F = 8.733, p* < 0.001.				SEE = 9.467, F = 5.884, *p*^†^ < 0.001.			

B, unstandardized regression coefficient; SE, standard error; β, standardized regression coefficient; CI, confidence interval; IV, intravenous; R, multiple correlation coefficient; R^2^, coefficient of determination; adjusted R^2^, adjusted coefficient of determination; β, standardized regression coefficient; SEE, standard error of the estimate; IV × OFT, interaction term between IV fluid therapy and oral feeding type. F, F statistic;.

* *p* values were obtained from multivariable linear regression analysis. The dependent variable was bilirubin decline rate.

† *p-*values were derived from the multivariable linear regression model. The dependent variable was bilirubin decline percentage.

## 4 Discussion

In this study, we evaluated the effects of IV fluid supplementation and enteral feeding patterns on bilirubin clearance in healthy term neonates undergoing intensive phototherapy for indirect hyperbilirubinemia. The main findings indicate that, after adjustment for baseline clinical differences, IV fluid supplementation was not independently associated with the rate or percentage of bilirubin decline, or the 6-h TSB levels. Conversely, mixed feeding was associated with faster bilirubin clearance. Baseline TSB level was observed to be a significant predictor of bilirubin decline, whereas maternal age was independently associated with both percentage and rate of decline. These findings suggest that the quality and adequacy of enteral intake, rather than hydration support alone, may play a more important role in the phototherapy response.

### 4.1 The effect of intravenous fluid supplementation on the bilirubin response

The clinical value of routine IV fluid supplementation as an adjunct to phototherapy for neonatal indirect hyperbilirubinemia remains controversial. Although some studies have suggested that additional fluid therapy may result in a limited reduction in TSB levels, particularly in the early treatment period, evidence remains inconsistent regarding whether it clinically significantly accelerates bilirubin decline or reduces phototherapy duration in healthy term neonates (3, 8–10, 21). Our findings are consistent with the literature and indicate that, when intergroup differences such as baseline TSB levels and postnatal age are considered, intravenous fluid supplementation does not make an independent contribution to the bilirubin response.

A clinically relevant aspect of this finding is that our study assessed the early response window of phototherapy. Previous evidence suggests that the potential effect of IV fluid supplementation may be most apparent within the first 4–8 h of treatment (11). The use of the 6-h TSB measurement in our study provided an opportunity to assess the extent to which this early effect could be observed in practice. Nevertheless, intravenous fluid supplementation was not identified as a significant predictor in multivariate analyses. On the other hand, according to the guidelines of the AAP, effective intensive phototherapy is expected to reduce TSB levels by 0.5–1 mg/dL per hour. The average bilirubin clearance rate of 0.83 mg/dL/hour observed in our study in infants fed exclusively via the enteral route, demonstrates that the AAP's effective phototherapy targets can be achieved through enteral feeding alone, without the need for additional IV fluid supplementation. This finding supports the aspect that routine IV fluid supplementation is not necessary in cases without clinical dehydration.

In our study, the higher 6-h TSB levels observed in the IV fluid supplementation group in unadjusted analyses may initially appear to indicate lower treatment efficacy. However, this finding is more likely related to a clinical indication bias. In routine clinical practice, IV fluid supplementation is rarely administered randomly; instead, it is generally preferred for infants with higher initial bilirubin levels or in those considered to be at greater clinical risk (16). Consequently, the difference observed in the unadjusted analyses probably reflects grater baseline clinical severity rather than true treatment effect. This interpretation is further supported by the absence of an independent association between IV fluid supplementation and bilirubin response in our extended multivariable sensitivity models.

Current guidelines recommend maintaining adequate enteral nutrition and hydration rather than routine IV fluid supplementation in phototherapy receiving neonates. IV fluid supplementation appears to be an approach that should be reserved for selected cases involving clinical dehydration, inadequate enteral intake, electrolyte imbalance, or more advanced clinical risk scenarios. Although, no patient in our cohort required a blood transfusion and no acute complications related to the intravenous catheter were observed, when indirect potential risks such as disruption of mother-infant interaction and increased hospital resource utilization are considered, there is no strong justification for the use of routine IV fluid supplementation.

### 4.2 The effect of feeding type and maternal Age on bilirubin elimination

Our study demonstrated that a mixed feeding regimen, in which formula milk is supplemented with breast milk, is independently associated with both the rate and percentage of bilirubin decline. This finding is consistent with the central role of enteral feeding in bilirubin metabolism. Possible mechanisms underlying this association include the fact that more effective enteral intake increases intestinal motility, accelerates the passage of meconium and stool, and thereby reduces the enterohepatic circulation of bilirubin. Consistent with this, previous studies have reported a slower phototherapy response in exclusively breastfed infants compared with those receiving mixed or formula feeding, whereas formula supplementation provides a faster biochemical response (15, 16, 22).

However, this finding should be interpreted with caution. As net enteral volume and calorie intake were not measured in our study, it remains open to debate whether the observed effect is attributable solely to the type of feeding or also to an increase in total enteral intake. However, a recent study reported that the amount of breast milk consumed by neonates receiving phototherapy had no statistically significant effect on the rate of bilirubin decline (*p* = 0.100) (23). Our findings should therefore not be interpreted as evidence directly supporting routine formula supplementation, but rather as an indication of how critical the nature of enteral intake is for bilirubin control, rather than the route of hydration.

From a clinical practice perspective, this point is particularly important. Formula supplementation may accelerate the reduction in bilirubin levels in the short term; however, the potential adverse effects this may have on the continuation of breastfeeding must also be considered. Indeed, it has been reported that the routine use of formula during phototherapy may adversely affect the rates of exclusive breastfeeding in the long-term following discharge (24). Given the immunological, metabolic and developmental benefits of breast milk, it would appear more appropriate to prioritize effective breastfeeding support, lactation counselling and the provision of adequate enteral hydration in the management of hyperbilirubinemia (25). Formula support should be considered as an individualized and temporary option in selected cases where breast milk is insufficient, weight loss is increasing, or enteral intake cannot be temporarily maintained at the target level.

Although our study demonstrated an independent positive association between maternal age and percentage decrease in bilirubin levels, this finding should be interpreted with caution. It is highly likely that maternal age represents unmeasured social and behavioral variables—such as breastfeeding experience, adherence to treatment recommendations, conscious adaptation to lactation management, family support, or care arrangements—rather than a direct biological effect (26). Therefore, whilst the finding regarding maternal age is clinically interesting, it should be regarded as hypothesis-generating.

The finding that the baseline TSB level is independently associated with the decline in bilirubin levels is fully consistent with the known kinetics of phototherapy. In cases where the baseline bilirubin load is higher, it is to be expected that more pronounced absolute decreases will be observed, particularly in the early stages, as the absorption of light energy increases. However, the fact that the baseline TSB level is included in the model both as an explanatory variable and indirectly in the calculation of certain clinical outcomes (rate and percentage of decline) requires a certain degree of caution in interpreting this relationship.

### 4.3 Strengths and limitations of the study

One of the main strengths of this study is that it sought to address, analytically, the issue of indication bias, which is frequently encountered in daily clinical practice. In observational studies, the more frequent use of IV fluid supplementation in infants with higher bilirubin levels or a higher-risk clinical presentation can create baseline imbalance between groups. In our study, we aimed to minimize these confounding effects by using extended multivariate regression and sensitivity analyses, thereby seeking to evaluate the independent associations between IV fluid supplementation and the type of enteral feeding in a more objective manner. Furthermore, the fact that both IV fluid supplementation and the type of feeding were examined together within the same cohort enhances the clinical applicability of the study.

However, there are important limitations that must be considered when interpreting our results. Firstly, the retrospective and single-center design of the study limits its ability to draw causal inferences and its generalizability. Furthermore, as the study is based on a single-center Turkish cohort, the findings may not be entirely applicable to different healthcare systems with varying clinical protocols, resource availability, or diverse demographic populations. Secondly, as the decision to initiate IV fluids was based on clinical judgement, confounding factors cannot be entirely ruled out despite multivariate adjustments. Thirdly, as the net volume, caloric content and feeding efficiency of enteral nutrition could not be objectively measured, the independent contributions of feeding type and total enteral intake could not be distinguished. Although a recent study reported that the amount of breast milk consumed had no statistically significant effect on the rate of bilirubin decline (23), this can still be considered a methodological limitation of our study. Lastly, as the study focused on the early bilirubin response, rebound hyperbilirubinemia, total phototherapy duration, rates of exclusive breastfeeding after discharge (17), and long-term neurodevelopmental outcomes could not be assessed. Nevertheless, evaluating the early 6-h TSB response holds significant clinical value, as it provides critical initial feedback on phototherapy efficacy and helps guide rapid clinical decisions to prevent escalation to invasive procedures such as exchange transfusion.

Furthermore, the fact that the baseline TSB level is both a key variable influencing clinical decisions and may be associated with clinical outcomes derived from bilirubin decline presents a methodological consideration in the form of mathematical coupling in the interpretation of the analyses. However, adjusting for baseline TSB was methodologically essential in our multivariable models to account for disease severity and to effectively mitigate the primary indication bias for IV fluid administration. Finally, as the cohort consisted solely of healthy term neonates, our findings should not be generalized to preterm infants, low birth weight infants, or neonates with significant dehydration, who may derive clinical benefit from IV fluid supplementation due to increased insensible fluid losses (9, 10, 27).

In addition, although the extended regression models were statistically significant, their explanatory power was modest, with adjusted R^2^ values of 0.167 for bilirubin decline rate and 0.112 for bilirubin decline percentage. Therefore, these models should not be interpreted as comprehensive predictive models of bilirubin elimination, but rather as exploratory models identifying independent associations. The modest explanatory power also suggests that additional unmeasured factors, such as actual enteral intake volume, breastfeeding efficiency, stool frequency, exact phototherapy exposure time, and individual bilirubin kinetics, may have contributed to bilirubin decline.

## 5 Conclusion

In conclusion, this study demonstrated that routine IV fluid supplementation, when added to intensive phototherapy in healthy term neonates with severe indirect hyperbilirubinemia, was not independently associated with the rate of bilirubin decline, the percentage reduction in bilirubin levels, or early-phase bilirubin response. In contrast, enteral feeding type, particularly mixed feeding with formula supplementation in addition to breast milk, was found to be associated with faster bilirubin clearance; although this finding should be interpreted cautiously given the heterogeneity in supplementation amounts.

These findings suggest that in the management of hyperbilirubinemia in healthy term neonates without clinical signs of dehydration, these observations support that routine IV fluid supplementation may not be necessary, and priority should be given not to routine IV fluid supplementation, but to effective phototherapy, adequate enteral hydration and robust lactation support. During treatment, formula supplementation may be considered as a temporary option to accelerate the response to phototherapy in cases where there is a medical necessity for breast milk to be insufficient; however, the clinical balance between the short-term biochemical benefit of this approach and long-term breastfeeding success must be carefully monitored. To validate these findings and ensure they are more robustly reflected in clinical guidelines, there is a need for multicenter, randomized controlled prospective studies comparing different feeding and hydration strategies.

## Data Availability

The raw data supporting the conclusions of this article will be made available by the authors, without undue reservation.
